# Interference With Quorum-Sensing Signal Biosynthesis as a Promising Therapeutic Strategy Against Multidrug-Resistant Pathogens

**DOI:** 10.3389/fcimb.2018.00444

**Published:** 2019-02-05

**Authors:** Osmel Fleitas Martínez, Pietra Orlandi Rigueiras, Állan da Silva Pires, William Farias Porto, Osmar Nascimento Silva, Cesar de la Fuente-Nunez, Octavio Luiz Franco

**Affiliations:** ^1^Programa de Pós-Graduação em Patologia Molecular, Universidade de Brasília, Brasília, Brazil; ^2^Centro de Análises Proteômicas e Bioquímicas, Universidade Católica de Brasília, Brasília, Brazil; ^3^S-Inova Biotech, Programa de Pós-Graduação em Biotecnologia, Universidade Católica Dom Bosco, Campo Grande, Brazil; ^4^Porto Reports, Brasília, Brazil; ^5^Synthetic Biology Group, MIT Synthetic Biology Center, Massachusetts Institute of Technology, Cambridge, MA, United States; ^6^Research Laboratory of Electronics, Massachusetts Institute of Technology, Cambridge, MA, United States; ^7^Department of Biological Engineering, Department of Electrical Engineering and Computer Science, Massachusetts Institute of Technology, Cambridge, MA, United States; ^8^Broad Institute of MIT and Harvard, Cambridge, MA, United States; ^9^The Center for Microbiome Informatics and Therapeutics, Cambridge, MA, United States

**Keywords:** virulence, antibiotic resistance, quorum sensing, quorum-sensing inhibition, anti-virulence therapy

## Abstract

Faced with the global health threat of increasing resistance to antibiotics, researchers are exploring interventions that target bacterial virulence factors. Quorum sensing is a particularly attractive target because several bacterial virulence factors are controlled by this mechanism. Furthermore, attacking the quorum-sensing signaling network is less likely to select for resistant strains than using conventional antibiotics. Strategies that focus on the inhibition of quorum-sensing signal production are especially attractive because the enzymes involved are expressed in bacterial cells but are not present in their mammalian counterparts. We review here various approaches that are being taken to interfere with quorum-sensing signal production via the inhibition of autoinducer-2 synthesis, PQS synthesis, peptide autoinducer synthesis, and N-acyl-homoserine lactone synthesis. We expect these approaches will lead to the discovery of new quorum-sensing inhibitors that can help to stem the tide of antibiotic resistance.

## Introduction

The increase in bacterial resistance to antimicrobial compounds and the spread of drug-resistant pathogens have become serious threats to human health. Currently, most antimicrobial compounds target essential bacterial physiological processes, thereby exerting a strong selective pressure on bacteria and facilitating the emergence and dissemination of resistant strains (Munguia and Nizet, 2017). Therapeutic strategies that circumvent the emergence and spread of multidrug-resistant pathogens are, therefore, urgently needed.

New attractive approaches for generating new therapeutics have focused on interfering with bacterial virulence factors, specifically, interfering with compounds synthesized by pathogens that facilitate colonization of the host and subsequent infection (Kong et al., 2016; Vale et al., 2016; Dickey et al., 2017; Munguia and Nizet, 2017). Because interference with virulence factors does not aim to eradicate the bacteria, it does not exert a strong selective pressure on the bacteria and probably decelerates the emergence and dissemination of resistant mutant strains (Gutierrez et al., 2009; Sully et al., 2014; Daly et al., 2015; Quave et al., 2015). However, the emergence of anti-virulence drug-resistant pathogens has been reported (Maeda et al., 2012; García-Contreras et al., 2013, 2015). Anti-virulence therapy appears all the more advantageous if we also consider that the production of virulence factors is under the control of regulatory mechanisms (e.g., quorum-sensing), and it should be possible to interfere with these mechanisms, consequently affecting the production of multiple virulence factors (Dickey et al., 2017; Defoirdt, 2018).

Quorum-sensing networks allow bacterial communication through the action of small diffusible autoinducer molecules (AI). These AI molecules comprise a diversity of molecular species such as autoinducer-2 (AI-2), acylated homoserine lactones (acyl-HSLs), oligopeptides, the *Pseudomonas* quinolone signal molecule (PQS), diffusible signal factor (DSF), γ-butyrolactone, 2-(2-hydroxyphenyl)-thiazole-4-carbaldehyde (IQS) among others (Guo et al., 2013; LaSarre and Federle, 2013; Pereira et al., 2013). Quorum-sensing systems operate in a cell density-dependent fashion, allowing the increase of AI concentration when cell density increases. After the AI concentration reaches a certain threshold, it triggers signaling events that modulate the expression of genes related to bacterial physiology, virulence, and biofilm formation (Papenfort and Bassler, 2016).

Interference with quorum-sensing systems has been envisioned as a suitable strategy to address the multi-drug resistance problem (Hirakawa and Tomita, 2013; Defoirdt, 2018). In this regard, a great diversity of compounds that interfere with quorum-sensing systems have been reported, as well as tools for their discovery (Jian and Li, 2013; Quave and Horswill, 2013; Nandi, 2016; Ali et al., 2017; Asfour, 2018). Strategies for inhibiting quorum sensing systems are designed mainly to interfere with the biosynthesis of AI, extracellular accumulation of the AI, and signal detection (LaSarre and Federle, 2013; Reuter et al., 2016; Singh et al., 2016; Haque et al., 2018). One of the most thoroughly explored strategies so far is interference with the extracellular accumulation of the signal. This interference can be achieved by using enzymes that degrade the signal or modify it, the use of antibodies that sequester the signal, as well as by synthetic polymers that sequester the signal (Fetzner, 2015; Daly et al., 2017; Ma et al., 2018). Interference in signal detection implies the use of compounds that interfere with the signal binding to the receptor (Singh et al., 2016; Wang and Muir, 2016; Kim et al., 2018). Other quorum-quenching strategies involve interfering with transcription factors binding to DNA and inhibiting the synthesis of the quorum-sensing signal (Gutierrez et al., 2009; Baldry et al., 2016; Scoffone et al., 2016; Greenberg et al., 2018).

The bacterial enzymes involved in quorum-sensing signal biosynthesis may be an attractive target for the development of anti-virulence agents because these enzymes are absent in mammals (Sun et al., 2004; Christensen et al., 2013; Pereira et al., 2013; Chan et al., 2015; Ji et al., 2016). Moreover, the inhibition of some of these enzymes could affect the production of more than one signal (Singh et al., 2006; Gutierrez et al., 2007, 2009; LaSarre and Federle, 2013). Experimental evidence suggests that dysfunctional AI-producing enzymes could turn pathogens less virulent for the host than pathogens expressing wild-type enzymes (Gallagher et al., 2002; Déziel et al., 2005; Kim et al., 2010; Komor et al., 2012). Thus, inhibiting the biosynthesis of the quorum-sensing signal could be a suitable strategy for developing anti-virulence agents. Because signal biosynthesis inhibition has emerged as an especially attractive way to perturb quorum-sensing networks, this strategy is emphasized in this review. The array of quorum-sensing signal biosynthesis inhibitors that have been developed, their main targets, the effects of these inhibitors on pathogen virulence, and new approaches for quorum-sensing signal biosynthesis inhibition will be summarized.

## Inhibition of Autoinducer-2 Synthesis

AI-2 compounds have been claimed as “universal” signal molecules involved in inter- and intra-bacterial species communication. This is supported by the fact that *luxS* gene homologs are widely distributed among bacterial genomes [*luxS* encodes the S-ribosylhomocysteine lyase (LuxS) enzyme, which synthesizes AI-2] (Pereira et al., 2013; Pérez-Rodríguez et al., 2015; Kaur et al., 2018). Moreover, some bacteria that are unable to produce AI-2 (e. g., *Pseudomonas aeruginosa* and *Riemerella anatipestifer*) respond to AI-2 external supply, and AI-2 mediates the interaction between polymicrobial biofilm members (Han et al., 2015; Li et al., 2015; Laganenka and Sourjik, 2017). In addition to regulation of biofilm formation, AI-2 has been linked to the regulation of pathogen virulence factors production, colonization capacity, persistence, and adaption to host environment (Armbruster et al., 2009; Li et al., 2017; Ma Y. et al., 2017). Therefore, interference with AI-2 production could be used as a strategy to attenuate pathogen virulence. Two main enzymes participate in AI-2 biosynthesis: Methylthioadenosine/S- adenosylhomocysteine nucleosidase (MTA/SAH nucleosidase) and LuxS. Both enzymes are involved in the activated methyl cycle, and they therefore influence bacterial metabolism. Strategies focused on inhibiting AI-2 production have, therefore, targeted these enzymes (Lebeer et al., 2007; Parveen and Cornell, 2011; LaSarre and Federle, 2013; Pereira et al., 2013).

## Methylthioadenosine/S-Adenosylhomocysteine Nucleosidase Inhibitors

**Figure 1:**
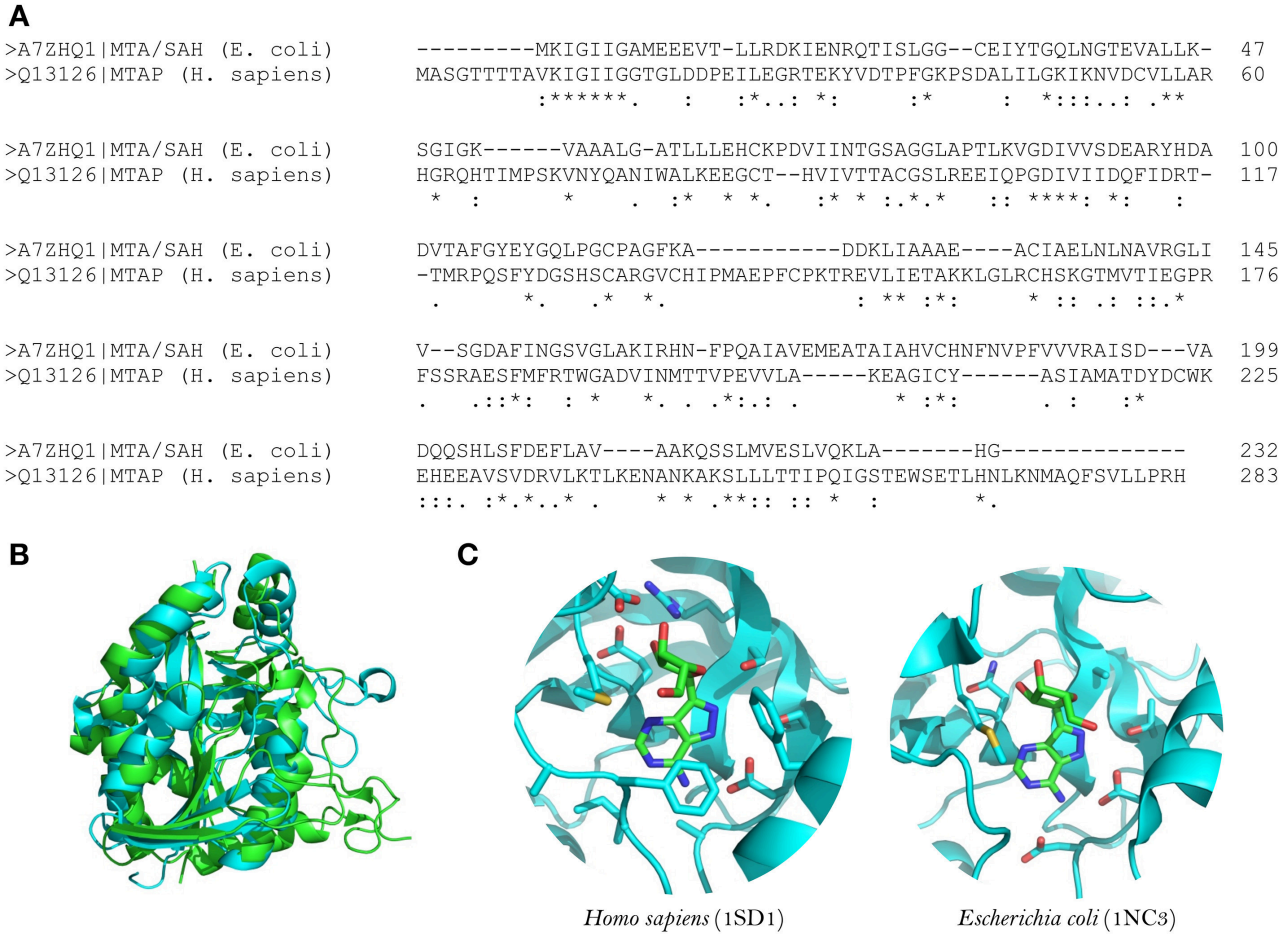
Structural comparison between human MTA phosphorylase and *E. coli* MTA/SAH nucleosidase. **(A)** Sequence alignment between *E. coli* MTA/SAH nucleosidase (MTAN; Uniprot ID: A7ZHQ1) and human MTA phosphorylase (MTAN; Uniprot ID: Q13126). **(B)** Structural alignment between MTAN (green) and MTAP (blue). **(C)** Active site comparison between MTAN (PDB ID: 1SD1, left) and MTAP (PDB ID: 1NC3, right) with formycin. The structures were retrieved from PDB, and the visualization was done by using Pymol v.1.6.

MTA/SAH nucleosidase has been identified in several bacterial species but is absent from mammalian cells (Sun et al., 2004). It is also linked to the acyl-HSLs biosynthesis pathway; therefore, MTA/SAH nucleosidase inhibition could interfere with the production of these quorum-sensing signals (Singh et al., 2006; Gutierrez et al., 2007, 2009). In addition, MTA/SAH nucleosidase appears to influence pathogens' capacity to produce biofilms (Bao et al., 2015; Han et al., 2017). Therefore, MTA/SAH nucleosidase could be an excellent choice as a target for the development of new quorum-sensing inhibitors. However, caution must be exercised, because the inhibition of MTA/SAH nucleosidase could result in the accumulation of S-adenosyl-homocysteine (SAH) and 5-methylthioadenosine (MTA) which, if present at high levels, could inhibit reactions catalyzed by polyamine synthases and S-adenosylmethionine dependent methyltransferases, interfering with bacterial growth (Heurlier et al., 2009; Parveen and Cornell, 2011). *Vibrio cholerae* MTA/SAH nucleosidase mutants with impaired growth have been reported (Silva et al., 2015). Nevertheless, experimental evidence has demonstrated that it is possible to inhibit MTA/SAH nucleosidase activity without severely affecting bacterial growth and without inducing resistance toward inhibitors (Gutierrez et al., 2009). In addition, Bourgeois et al. (2018) observed that a *Salmonella enterica* serovar Typhimurium Δ*metJ* mutant strain, which was defective in methionine metabolism, presented elevated intracellular MTA levels without affecting bacterial growth (Bourgeois et al., 2018). In a *S. aureus pfs* mutant strain (*pfs* encodes the MTA/SAH nucleosidase), growth was not impaired in nutrient-rich conditions but it was affected in zebrafish embryos (Bao et al., 2013). MTA is also a substrate of the human enzyme MTA phosphorylase, but the structural differences between the human and bacterial enzymes (in the purine, ribose and 5′-alkylthio binding sites) make it possible to develop MTA structural analogs as inhibitors that are selective for MTA/SAH nucleosidase (Lee et al., 2004; Guo et al., 2013; Figure 1).

The structures of MTA/SAH nucleosidase homologs in several bacterial species have been resolved; these species include *Escherichia coli, Helicobacter pylori, Streptococcus pneumoniae, Staphylococcus aureus, S. enterica, V. cholerae, Brucella melitensis*, and more recently *Aeromonas hydrophila* (Lee et al., 2003; Singh et al., 2006; Siu et al., 2008; Ronning et al., 2010; Haapalainen et al., 2013; Kang et al., 2014; Thomas et al., 2015; Xu et al., 2017). Basically, the MTA/SAH nucleosidase is a homodimer that contains two active sites in which specific sub-sites (purine, ribose and 5′-alkylthio binding sites) are involved in the interactions with the substrate (Ronning et al., 2010). MTA/SAH nucleosidase removes adenine from SAH, MTA, and 5′-deoxyadenosine yielding S-ribosyl-L-homocysteine (SRH), S-methyl-5′-thioribose, and 5′-deoxyribose, respectively. In *Neisseria meningitidis* and *H. pylori*, this reaction takes place through an early transition state while in other bacteria such as *Klebsiella pneumoniae, E. coli, S. aureus*, and *S. pneumoniae* it occurs through a late dissociative transition state (Singh et al., 2005b; Gutierrez et al., 2007). In line with this, most of the MTA/SAH nucleosidase inhibitors that have been developed are transition state analogs (Singh et al., 2005a,b, 2006; Gutierrez et al., 2007, 2009).

Several of these transition state analogs have been tested on *E. coli* and *S. pneumoniae* MTA/SAH nucleosidases (Singh et al., 2005a, 2006). The analogs were mainly based on 5′-thio-Immucillin-A and 5′-thio-DADMe-Immucillin-A derivate compounds, in which diverse chemical groups (aromatic, cycloalkyl, halogenated aliphatic and hydrophobic groups) were incorporated at the 5′-thio position. The 5′-thio-DADMe-Immucillin-A-derived analogs behaved as more potent MTA/SAH nucleosidase inhibitors than 5′-thio-Immucillin-A-derived analogs, because these analogs mimic the late dissociative transition state through which the enzymatic reaction in these bacterial species takes place (Singh et al., 2005a, 2006). Moreover, 5′-thio-DADMe-Immucillin-A-based inhibitors showed *V. cholerae* N16961 and *E. coli* O157: H7 cellular MTA/SAH nucleosidase inhibition. The inhibitors interfered with AI production without affecting bacterial growth. Although the inhibitors reach their intracellular target, a significant diffusion barrier was observed. For both *V. cholerae* and *E. coli*, the inhibition of AI-2 production by 5′-butylthio-DADMe-Immucillin-A was sustained over several bacterial generations, suggesting that bacterial resistance had not emerged toward the MTA/SAH nucleosidase inhibitor. The 5′-butylthio-DADMe-Immucillin-A inhibitor reduced biofilm production in both species of bacteria (Gutierrez et al., 2009). However, Silva et al. (2015) recently showed that *V. cholerae* N16961 was able to form biofilm when treated with 5′-methylthio-DADMe-Immucillin-A, although a high percentage of MTA/SAH nucleosidase inhibition was reached. In addition, in two MTA/SAH nucleosidase mutant strains biofilm production was similar to the wild-type strain (*V. cholerae* N16961) whereas the growth rate and swarming motility were lower than the wild-type strain (Silva et al., 2015).

In another line of research, novel inhibitors of *S. enterica* MTA/SAH nucleosidase based on transition state analogs were designed. Interestingly, *S. enterica* MTA/SAH nucleosidase presented an elongated 5′-alkylthio-binding pocket. In that case, the design of novel inhibitors involved adding elongated 5′-alkylthio groups to the DADMe-Imm-A core to fill this site. The new inhibitors were 2-hydroxyethylthio-DADMe-Imm-A, 3-hydroxypropylthio-DADMe-Imm-A, 4-hydroxybutylthio-DADMe-Imm-A and 2-(2-hydroxyethoxy)ethylthio-DADMe-Imm-A, all of which showed dissociation constants in the pM range (Haapalainen et al., 2013). Recently, the putative *Mycobacterium tuberculosis* MTA/SAH nucleosidase (Rv0091) was expressed and characterized kinetically, showing a preference for 5′-deoxyadenosine as the substrate in comparison with MTA and SAH. For Rv0091, DADMe-Imm-A inhibitors consisting of derivatized analogs with long alkyl groups at the C5′ position exerted potent inhibitory activity (Namanja-Magliano et al., 2016). Additionally, when using 5′-deoxyalkyl- and 5′-alkylthio-DADMe-Immucillin-A transition state analogs it was observed that for the 5′-deoxyalkyl-DADMe-Immucillin-A analogs, shorter 5′-alkyl-substituents led to the most potent inhibition, in contrast to 5′-alkylthio-DADMe-Immucillin-A analogs, in which longer 5′-alkyl-substituents led to the most potent inhibition. These inhibitors did not affect the growth of *M. tuberculosis* or *Mycobacterium smegmatis*; however, they showed an antimicrobial effect on *H. pylori* (due to the involvement of *H. pylori* MTA/SAH nucleosidase in the menaquinone biosynthesis pathway). The authors suggested that Rv0091 plays a role in 5′-deoxyadenosine recycling but is not essential for the growth of *M. tuberculosis* or *M. smegmatis* (Namanja-Magliano et al., 2017).

Furthermore, the MTA/SAH nucleosidase has been suggested to influence the virulence of pathogens in an AI-2-independent fashion. Based on mouse infection models and zebrafish embryo infection models, Bao et al. (2013) demonstrated that a *S. aureus* NCTC 8325 *pfs* mutant strain displayed attenuated virulence *in vivo*. In addition, a *luxS* mutant was as virulent as the isogenic wild-type *S. aureus* NCTC8325 strain, suggesting that the effects of *pfs* deletion on *S. aureus* virulence were independent of the AI-2-based quorum sensing pathway. The attenuated virulence of the *pfs* mutant strain was associated with reduced proliferation *in vivo*. Additionally, *in vitro* analysis showed reduced extracellular protease activity in the *pfs* mutant strain linked to reduced *sspABC* operon transcription and *aur* gene transcription (Bao et al., 2013). Another study showed that *S. aureus* NCTC 8325 *pfs* mutant strain displayed reduced biofilm formation *in vitro* by AI-2-independent mechanisms. The *pfs* deletion reduced the transcription of autolysis-related genes *atlE* and *lytM* in the mutant strain; therefore, autolysis-dependent extracellular DNA release in the *pfs* mutant, and consequently biofilm formation, was affected (Bao et al., 2015).

The experimental findings reviewed above suggest that MTA/SAH nucleosidase could influence pathogen virulence via quorum-sensing-independent or –dependent mechanisms (Gutierrez et al., 2009; Bao et al., 2013, 2015; Silva et al., 2015). Therefore, for MTA/SAH nucleosidase inhibitor evaluation studies, precise experimental designs aimed at distinguishing by which mechanism (i.e., quorum sensing-independent and/or –dependent) the pathogen's virulence is affected are warranted. Moreover, most of the studies about MTA/SAH nucleosidase inhibition have been focused on designing inhibitors and evaluating their inhibitory activity via enzymatic assays using purified enzymes, but data about the real impact that such inhibitors have on pathogens virulence is scarce (Singh et al., 2005a,b, 2006; Gutierrez et al., 2007; Haapalainen et al., 2013; Namanja-Magliano et al., 2016).

Therefore, it is essential to perform studies that evaluate the effects of MTA/SAH nucleosidase inhibitors on pathogen virulence gene expression as well as testing the effectiveness of these to attenuate pathogens *in vivo*. Immucillin-based inhibitors appear to be a reasonable option as quorum quenching agents. This class of inhibitors has been used as antiviral, antibacterial (specifically in *H. pylori*), anti-malarial, and antineoplastic agents. Some of them are in clinical trials or have been approved for use in humans. Immucillin-based inhibitors are chemically stable, specific, and it is possible to chemically modify them to gain bioavailability without severely affecting their inhibitory activity (Longshaw et al., 2010; Evans et al., 2018).

## S-Ribosylhomocysteine Lyase Inhibitors

The enzyme S-ribosylhomocysteine lyase (LuxS) is a potential target for the development of new therapeutic agents because it is present in numerous bacterial species but not in mammals (Pereira et al., 2013; Pérez-Rodríguez et al., 2015; Kaur et al., 2018). In addition, LuxS also appears to modulate bacterial biofilm formation based on results obtained with *luxS* mutant bacteria (Hardie and Heurlier, 2008; Kang et al., 2017; Ma R. et al., 2017; Zuberi et al., 2017). However, through which mechanisms *luxS* influences biofilm formation is under debate. LuxS could influence biofilm formation in an AI-2-dependent fashion, in which the expression of genes associated with bacterial adherence and biofilm matrix production may be modulated through AI-2-mediated signaling (Hardie and Heurlier, 2008; Duanis-Assaf et al., 2016; Ma R. et al., 2017; Velusamy et al., 2017; Pang et al., 2018). In addition, AI-2 may promote single and mixed species biofilm formation through chemotaxis-mediated aggregation events (Laganenka et al., 2016; Laganenka and Sourjik, 2017). However, other findings suggest that *luxS* also influences biofilm formation in an AI-2 signaling-independent fashion, probably involving the activated methyl cycle, fimbriation modulation and biofilm-associated gene expression modulation (Niu et al., 2013; Hu et al., 2018; Yadav et al., 2018).

Furthermore, LuxS may influence the virulence of pathogens during the host infection process. Recently, Yadav et al. (2018) demonstrated using a rat model of otitis media that *S. pneumoniae* D39Δ*luxS* mutant strain had decreased capacity for host colonization in comparison with the wild-type strain. Similarly, in a murine model D39Δ*luxS* displayed reduced capacity of nasopharynx colonization as well as reduced dissemination toward lung and blood. Interestingly, when AI-2 was administered to the D39Δ*luxS* infected mice, the D39Δ*luxS* mutant became as virulent as the wild-type strain without AI-2 treatment, suggesting that attenuated virulence in D39Δ*luxS* was associated with impaired AI-2 production and signaling (Trappetti et al., 2017). Moreover, in mice dual-infected with wild-type *Borrelia burgdorferi* and a *luxS* mutant strain, a higher wild-type bacterial load was observed than *luxS* mutant bacterial load in distal tissues from infection site, suggesting attenuated virulence for the *luxS* mutant (Arnold et al., 2015). However, using a pneumonic plague mouse model Fitts et al. (2016) observed that a *Yersinia pestis* CO92 Δ*luxS* mutant was as virulent as the wild-type CO92 strain. In addition, deletion of l*uxS* from a Δ*rbsA*Δ*lsrA* strain (attenuated virulence) turns it into the Δ*rbsA*Δ*lsrA*Δ*luxS* mutant, which was also as virulent as the wild-type strain (Fitts et al., 2016).

Taking into consideration the experimental evidence mentioned above, implementation of therapeutic strategies focused on LuxS inhibition may turn out to be complex and more difficult than initially envisioned. The effects of *luxS* on biofilm formation and virulence appear be dependent on bacterial species and genetic background (Bao et al., 2013; Fitts et al., 2016; Ma Y. et al., 2017; Trappetti et al., 2017; Velusamy et al., 2017; Hu et al., 2018). Also, LuxS could influence gene expression in an AI-2 signaling-independent fashion (Pereira et al., 2013). Moreover, LuxS inhibition could facilitate the accumulation of toxic compounds that disturb bacterial viability, and it could therefore exert selective pressure on the pathogen (Heurlier et al., 2009). In addition, the effects of *luxS* suppression on bacterial growth could be dependent on environmental stress conditions (Park et al., 2017). LuxS-independent pathways could also be involved in AI-2 formation (Tavender et al., 2008). However, *in vivo* studies have shown that it is possible to attenuate pathogen virulence via LuxS inhibition (Zhang et al., 2009; Sun and Zhang, 2016).

**Figure 2:**
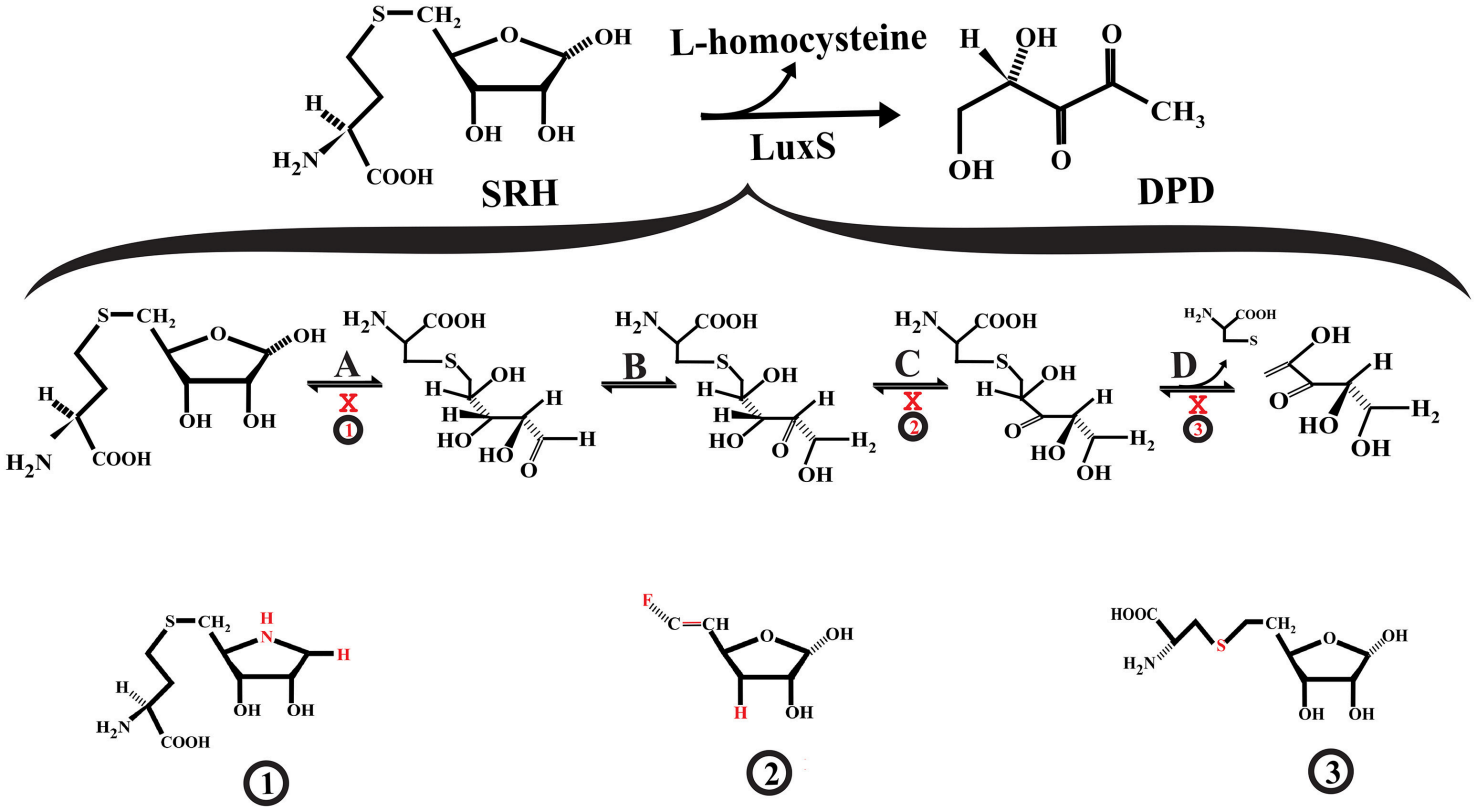
Interference with S-ribosylhomocysteine lyase (LuxS) activity. **(A)** S-ribosyl-homocysteine (SRH) ribose ring opening. **(B)** The opened SRH molecule undergoes aldose-ketose isomerization yielding 2-keto intermediate. **(C)** The 2-keto intermediate is transformed into 3-keto intermediate via ketose-ketose isomerization. **(D)** The 3-keto intermediate suffers a β-elimination reaction releasing L-homocysteine and the enol form of DPD. The SRH analog S-(1-Amino-1,4-anhydro-1,5-dideoxy-D-ribitol-5-yl)-L-homocysteine (1) may act as a competitive inhibitor that not suffer ring opening, affecting the aldose-keto isomerization. The 3,5,6-trideoxy-6-fluoro-D-erythro-hex-5-enofuranose (2) may interfere with the formation of the 3-keto intermediate. The S-homoribosyl-L-cysteine inhibitor (3) may interfere with the β-elimination step. The red X indicates inhibition.

LuxS is a homodimer metalloenzyme that catalyzes the SRH cleavage through a proposed mechanism involving two isomerization steps (aldo-ketose and keto-ketose isomerization) followed by a β-elimination step to yielding L-homocysteine and the enol form of 4,5 dihydroxy-2,3-pentanedione (DPD) (Zhu et al., 2003, 2006; Pei and Zhu, 2004; Figure 2). The DPD spontaneously cyclizes and reorganizes into various furanone molecules that constitute the AI-2 (LaSarre and Federle, 2013). In this regard, most of the strategies for directly inhibiting LuxS are centered on substrate analogs that compete with the natural substrate for binding to the enzyme active site and interfere with any of the mechanistic steps that LuxS catalyzes (Alfaro et al., 2004; Wnuk et al., 2008; Malladi et al., 2011; Figure 2).

Some of the reported LuxS competitive inhibitors are SRH analogs that contain a modified C1 at the ribose or [4-aza]ribose, which could affect the initial ring opening and the subsequent isomerization [e.g., S-anhydroribosyl-L-homocysteine, and S-(1-Amino-1,4-anhydro-1,5-dideoxy-D-ribitol-5-yl)-L-homocysteine] (Alfaro et al., 2004; Malladi et al., 2011; Figure 2, compound 1). Another potential LuxS inhibitor that could affect the ring opening is the SRH analog S-(1,5-Dideoxy-4-thio-D-ribofuranos-5-yl)-L-homocysteine (Sobczak et al., 2015). Moreover, compounds that interfere with the tautomerization/isomerization steps during the catalytic cycle act as competitive inhibitors of LuxS. Some of these compounds are SRH analogs in which the hydroxyl group at ribose C3 position has been removed or modified, making it a non-enolizable hydroxyl group and therefore interfering with the formation of the 3-ketone intermediate (e.g., 3,5,6-trideoxy-6-fluoro-D-erythro-hex-5-enofuranose) (Wnuk et al., 2008; Figure 2, compound 2). In addition, analogs of enediolate intermediates in which the enediolate moiety was substituted by a planar hydroxamate group act as powerful reversible competitive inhibitors of LuxS (Shen et al., 2006).

Inhibitors of the LuxS catalytic β-elimination step targeted the carbon (C5)-sulfur bond or the hydrogen atom at C4 position in the SRH substrate. In the inhibitor S-homoribosyl-L-cysteine, the carbon (C5)-sulfur bond was replaced by a C5-C6 carbon-carbon bond, which interferes with the cleavage of the carbon-sulfur bond (Alfaro et al., 2004; Figure 2, compound 3). Recently, Chbib et al. (2016) synthesized 4-C-Alkyl/aryl-SRH analogs that potentially could inhibit LuxS at the β-elimination step. The substitution of the hydrogen atom at C4 position by alkyl/aryl groups should prevent the abstraction of the C4-proton, which is important for the β-elimination step (Chbib et al., 2016). Interestingly, these authors suggested that the 4-C-Alkyl/aryl-SRH analogs also could inhibit LuxS by interfering with the dimerization of the enzyme. Previously, SRH analogs that carried alkyl/aryl groups at the C3 position of the homocysteine moiety of SRH were suggested as LuxS dimerization inhibitors (Liu, 2012).

Indeed, LuxS inhibitors [e.g., S-xylosylhomocysteine and S-(3-deoxy-3-fluoroxylosyl) homocysteine] that exerted a time-dependent inhibition of LuxS have been identified. They act as slow-binding inhibitors of improved potency, similarly to the halogenated S-[3-Bromo-3,5-dideoxy-D-ribofuranose-5-yl]-L-homocysteine and S-[3-fluoro-3,5-dideoxy-D-ribofuranos-5-yl]-L-homocysteine analogs (Gopishetty et al., 2009; Wnuk et al., 2009). The time-dependent inhibition exerted by these halogenated analogs was produced by the enzyme-catalyzed elimination of halide ions (Gopishetty et al., 2009). Equally, time-dependent inhibition of LuxS was observed for the S-[4-Amino-4,5-dideoxy-α/β-D-ribofuranos-5-yl]-L-homocysteine hemiaminal analog. This time-dependent inhibition was suggested as a result from the formation of ketone intermediates that bind to the LuxS active site with a higher affinity than the ribose natural substrate (Malladi et al., 2011).

Molecules that covalently modify LuxS also mediate its inhibition. Along these lines, halogenated furanones have been shown to inactivate LuxS. Specifically, it was observed that furanones that contain a vinyl monobromide moiety inhibit LuxS in a concentration-dependent manner. Mechanistic studies showed that LuxS was inactivated by covalent modification (Zang et al., 2009). Recently, it was hypothesized that the 2-deoxy-2-propylthiol-S-ribosylhomocysteine potentially could inhibit LuxS via covalent modification (Chbib, 2017).

Most of these luxS inhibitors have been tested *in vitro* using enzymatic assays with the purified LuxS enzyme. However, if these inhibitors are able to inhibit luxS *in vivo* with the consequent attenuation of pathogen virulence still needs to be investigated.

In addition to the SRH substrate analogs described above, other LuxS inhibitors are substrate non-analogs. Using phage display, representative peptide sequences that bind to LuxS were found. Of these, only the peptide TNRHNPHHLHHV inhibited LuxS and, then, only weakly, showing that there is not necessarily a correlation between peptide binding to LuxS and enzyme inhibition (Han and Lu, 2009). However, two LuxS-derived peptides have been described [peptide 5411 (MHTLEHLFAGFM) and 5906 (MLFAGFM)] that acted as potential LuxS inhibitors and mediated the attenuation of *Edwardsiella tarda* TX1 virulence *in vivo* (Zhang et al., 2009). The expression of the inhibitory peptides in TX1 strain (via plasmids) affected AI-2 production, reduced biofilm formation, and reduced *esrA* and *orf26* gene expression. Additionally, the virulence of TX1 strain was attenuated in infected Japanese flounder fish. Fish infected with the TX1 strain could be attenuated either by means of a commensal *Pseudomonas* sp. strain expressing the peptide 5411 or by directly expressing such peptide in tissues of infected fish (Zhang et al., 2009). Recent findings demonstrated that peptide 5906 production by *E. coli* DH5α/p5906 or by fish tissues attenuated *E. tarda* pathogenesis in Japanese flounder. In addition, the pathogenesis of *A. hydrophila* AH1 and *V. harveyi* T4 in infected fish was attenuated by *E. coli* DH5α/p5906. However, this attenuation was moderate in comparison with the attenuation that *E. tarda* underwent. It has been suggested that differences in the LuxS sequences of these pathogens could be responsible for the observed differences in the attenuation levels (Sun and Zhang, 2016). All these findings suggest that LuxS-derived peptides have the potential to act as AI-2-based quorum sensing system inhibitors in several bacterial species and that engineered commensal bacteria to produce LuxS-derived peptides could be a feasible strategy for quorum quenching *in vivo*.

To disturb LuxS functionality, the design of small molecule inhibitors has been the most exploited strategy. However, interference with the *luxS* gene expression represents an alternative approach to impairing LuxS function. The development of the Clustered Regularly Interspaced Short Palindromic Repeats-Cas 9 (CRISPR-Cas9) genome-editing technology now permits the expression of genes to be modulated with a high specificity and reduced off-target effect. This technology has been proposed as a promising method for fighting against antimicrobial resistance (Greene, 2018). Recently, Kang et al. (2017), using CRISPR-Cas9 genome-editing technology, obtained *E. coli* SE15Δ*luxS* mutant clones from the *E. coli* SE15 clinical strain isolated from the indwelling catheter of a patient suffering from urinary tract infections. *E. coli* SE15Δ*luxS* clones showed reduced biofilm formation in comparison to the wild-type strain. Moreover, in *E. coli* SE15Δ*luxS* mutant the expression of the genes *mqsR, pgaB, pgaC, csgE*, and *csgF* (involved in biofilm formation) was down-regulated (Kang et al., 2017). This work showed that by using CRISPR-Cas9 genome-editing technology it is possible to disturb the *luxS* gene expression and consequently impair one of the mechanisms of pathogenicity (biofilm formation) employed by pathogens. However, if this approach will be effective *in vivo* remains to be seen. Nevertheless, the feasibility of CRISPR-Cas9 genome-editing technology for attenuating the virulence of pathogens *in vivo* has been demonstrated. In this respect, the use of CRISPR-Cas9 phagemid vectors attenuated the virulence of enterohaemorrhagic *E. coli* in a *Galleria mellonella* infection model and *S. aureus* in a mouse skin colonization model (Bikard et al., 2014; Citorik et al., 2014).

An alternative CRISPR-Cas9-based approach, namely the CRISPR interference (CRISPRi) approach, has been applied successfully for *luxS* attenuation in clinical bacterial isolates (Zuberi et al., 2017). This system is based on the use of a Cas-9 DNA endonuclease that is not catalytically active (dCas-9) but can be directed toward the target gene by a small guide RNA and repress the expression of the target gene via interfering with the transcriptional process (Qi et al., 2013). Using a CRISPRi approach that targeted the *luxS* gene of the *E. coli* clinical strain AK-117 (isolated from urinary catheters), Zuberi et al. (2017) obtained three *luxS* knockdown strains (AK-LV1, AK-LV2, and AK-LV3) that were metabolically actives but with impaired biofilm formation capacity (Zuberi et al., 2017).

It has been described that in *E. coli* the CyaR small RNA regulated *luxS* gene expression negatively via post-transcriptional binding to the *luxS* mRNA 5′end (including the ribosome binding site), consequently, CyaR small RNA expression reduced AI-2 production (De Lay and Gottesman, 2009). Moreover, Zhang and Sun (2012) using an antisense RNA interference approach, impaired *luxS* expression in *Edwardsiella ictaluri* J901 strain, yielding the *luxS*-defective *E. ictaluri* J901Ri strain. *E. ictaluri* J901Ri showed lower AI-2 production, reduced biofilm formation, and down-regulated expression of *orf26, esrA, eseB, eseD, eihA*, and *wbiT* genes in comparison to *E. ictaluri* J901C control strain. Moreover, *E. ictaluri* J901Ri-infected zebrafish group showed lower accumulated mortality than *E. ictaluri* J901C- infected group, and *E. ictaluri* J901Ri infectivity on ZF4 cells was reduced in comparison to the control strain (Zhang and Sun, 2012). Therefore, it was possible that using an RNA interference approach attenuated the *E. ictaluri* J901 virulence. Based on an antisense RNA interference approach that targeted the *luxS* gene, Zhang et al. (2008), attenuated the virulence of the pathogen *E. tarda*. The *E. tarda* TX1/pJR18 strain, which contained a plasmid (pJR18) that constitutively expressed the *luxS* antisense RNA, showed lower AI-2 production, reduced biofilm formation and reduced expression of *esrA* and *orf26* genes in comparison to the control *E. tarda* TX1/pJRA strain. Furthermore, in *E. tarda* TX1/pJR18-infected Japanese flounder group the accumulated mortality, the bacteria recovered from blood and kidney, and *orf 26* and *esrA* expression were lower than the *E. tarda* TX1/pJRA-infected group (Zhang et al., 2008). Based on all these findings, it could be envisioned that antisense oligonucleotide-based inhibition could be a feasible strategy for the development of new luxS inhibitors.

## Inhibition of PQS Synthesis

*Pseudomonas aeruginosa* produces 2-heptyl-3-hydroxy-4(1H)-quinolone, which is commonly known as *Pseudomonas* quinolone signal (PQS) and acts as a quorum-sensing signal molecule (Déziel et al., 2004). Among the proteins involved at PQS synthesis, several of them are encoded by the *pqsABCDE* operon. The first step in the PQS biosynthesis pathway involves the formation of anthraniloyl-coenzyme A from anthranilate catalyzed by an anthranilate CoA ligase (PqsA). Subsequently, two condensation reactions take place. First, anthraniloyl-coenzyme A condenses with malonyl-CoA to form (2-aminobenzoyl) acetate with the participation of the proteins PqsD and PqsE. Second, the (2-aminobenzoyl) acetate condenses with octanoate through the PqsB and PqsC catalytic activity, producing 2-heptyl-4(1H)-quinolone (HHQ). Finally, HHQ is hydroxylated by PqsH FAD-dependent monooxygenase to form PQS (Déziel et al., 2004; Dulcey et al., 2013). Both HHQ and PQS act as signaling molecules.

Several enzyme-catalyzed reactions in the PQS biosynthesis pathway are being targeted for interference with PQS production (Sahner et al., 2013; Hinsberger et al., 2014; Ji et al., 2016; Maura et al., 2017). Anthranilate-CoA ligase (PqsA) constitutes an attractive target for developing drugs because the ortholog enzyme is absent in humans. PqsA catalyzes the conversion of anthranilate to anthraniloyl-coenzyme A in a reaction that involves an anthranilyl-AMP intermediate, which has been targeted in the design of PqsA inhibitors (Ji et al., 2016).

Recently, Ji et al. (2016) designed and evaluated the inhibitory activity of several sulfonyladenosine compounds on PqsA. These small molecules mimic the anthranilyl-AMP intermediate. The anthranilyl-AMS and anthranilyl-AMSN compounds were the most potent PqsA inhibitors that were found to reduce HHQ and PQS quinolone production by *P. aeruginosa* strain PA14; while salicyl-AMS, salicyl-AMSN, and benzoyl-AMS inhibitors were less potent. The authors suggested that differences in cell penetration, stability, and/or target specificity could be responsible for the variations in potency observed in inhibiting HHQ and PQS quinolone production (Ji et al., 2016). Other types of PqsA inhibitors that have been studied are the substrate analogs. Challenging the *P. aeruginosa* strain PAO1 culture with the anthranilate analog methyl-anthranilate was observed to inhibit the production of PQS and to decrease activity of the virulence factor elastase in a concentration-dependent manner. Interestingly, the methyl-anthranilate treatment did not affect the growth of cultures (Calfee et al., 2001). Moreover, other anthranilic acid analogs, specifically halogenated anthranilic acid analogs, exerted inhibitory activity on the production 4-hydroxy-2-alkylquinolines (HAQs) in *P. aeruginosa* and *Burkholderia thailandensis* without significantly disturbing bacterial growth (Lesic et al., 2007). The treatment of *P. aeruginosa* with several of these halogenated analogs represses the expression of HAQ biosynthetic operons *pqsA-E* and *phnAB* as well as the virulence factors pyocyanin (*phz ABCDEFG, phzH, phzM*, and *phzS*), hydrogen cyanide (*hcnABC*), chitinase (*chiC*), lectins (*lecA and lecB*), and elastase (*lasB*). Interestingly, these compounds are also effective *in vivo*, as they limited the virulence of *P. aeruginosa* in mice, delayed mortality in the treated animals, reduced the production of HHQ, and prevented systemic dissemination of the bacteria (Lesic et al., 2007). Furthermore, Coleman et al. (2008) tested the inhibitory activity of several anthranilate analogs on PQS production in bacterial cultures as well as on PqsA activity. Most of the chloro- and fluoro-anthranilate derivatives inhibited the production of PQS in *P. aeruginosa* culture and were PqsA substrates. Additionally, the anthranilonitrile, 5-nitroanthranilonitrile, methylanthranilate, and 3-fluoro-O-anisidine analogs did not behave as PqsA substrates but inhibited the production of PQS (Coleman et al., 2008).

Another protein involved in the PQS biosynthesis pathway that has been considered for the development of anti-virulence drugs is PqsD. PqsD forms homodimers in solution and structurally is similar to *E. coli* β-ketoacyl-ACP synthase III (FabH), showing the Cys-His-Asn catalytic triad typical of FabH-like enzymes (Bera et al., 2009). PqsD catalyzes the formation of 2-aminobenzoylacetyl-CoA in the PQS biosynthesis pathway using as substrates anthraniloyl-CoA and malonyl-CoA. Initially PqsD forms an anthraniloyl-PqsD intermediate via Cys 112 in the PqsD active site; subsequently, a condensation reaction takes place with malonyl-CoA. Given the structural similarity between PqsD and FabH-like enzymes, it has been suggested that FabH inhibitors potentially could inhibit PqsD (Pistorius et al., 2011; Dulcey et al., 2013).

Along the same lines, Pistorius et al. (2011) demonstrated that the well-established FabH inhibitors, such as 2-(4-bromo-3-diethylsulfamoyl-benzoylamino)-benzoic acid and 2-[(2- phenoxybiphenyl-4-carbonyl) amino] benzoic acid, had IC_50_ in the micromolar range; therefore, they exerted a modest inhibitory activity toward PqsD (Pistorius et al., 2011). Subsequently, introducing modifications in the 2-(4-bromo-3-diethylsulfamoyl-benzoylamino)-benzoic acid inhibitor yielded a series of sulfonamide-substituted benzamidobenzoic acids that inhibited PqsD. It was suggested that the binding of these compounds within the anthraniloyl-CoA channel of PqsD (involving hydrogen bonds, π-stackings, and hydrophobic interactions) hinders the access of substrate to the catalytic site; the compounds are therefore acting as entropy-driven channel-blocker inhibitors (Weidel et al., 2013). Moreover, Hinsberger et al. (2014) identified PqsD inhibitors with preferential selectivity to PqsD over RNA polymerase. These inhibitors were derivatives of benzamidobenzoic acid. The selectivity to PqsD was favored by introducing modifications on the benzamidobenzoic acid scaffold. The development of PqsD inhibitors that minimally affect the activity of RNA polymerase is desirable, because compounds that hinder this activity could exert selective pressure on the targeted bacteria (Hinsberger et al., 2014).

Using a ligand-based approach, Storz et al. (2012), identified several PqsD inhibitors that consisted of PqsD substrates and transition state analogs. The most potent inhibitor was (2-nitrophenyl)phenyl methanol, which contained a (2-nitrophenyl)methanol core rigidified by an unsubstituted phenyl moiety. This molecule inhibited the production of HHQ and PQS by *P. aeruginosa* PA14 cultures and reduced the biovolume of biofilm formed by this bacterial strain. At a concentration of 250 μM, this molecule did not affect bacterial growth or have a toxic effect on human THP-1 macrophages (Storz et al., 2012). A subsequent study showed that the inhibition of PqsD by (2-nitrophenyl)phenyl methanol was time-dependent (Storz et al., 2013). Subsequently, Storz et al. (2014) synthesized and evaluated several (2-nitrophenyl)methanol derivatives with improved *in vitro* PqsD inhibition. However, most of these derivatives did not show similar potency in inhibiting HHQ production in a *pqsH*-deficient *P. aeruginosa* PA14 strain. The derivative that contained an ethyl group at the methanol moiety, as well as those which contained heteroaromatic pentacycles, strongly inhibited PqsD activity in cells, even though these derivatives were not among the best PqsD inhibitors *in vitro*. Therefore, for (2-nitrophenyl)methanol derivatives, improved *in vitro* PqsD inhibition does not necessarily mean improved inhibitory activity *in cellulo* (Storz et al., 2014).

Other PqsD inhibitors that have been reported are compounds of the aryl-ureidothiophene-2-carboxylic acid class. These compounds were predicted to bind to the substrate channel of PqsD via their aryloxy-moiety pointed toward the bottom of the pocket and thereby block the binding of the substrate, anthraniloyl-CoA (Sahner et al., 2013). Moreover, the chemical structure combination of ureidothiophene-2-carboxylic acids with (2-nitrophenyl)methanol inhibitors yielded some derivatives with improved PqsD inhibitory activity when activity was measured in a cell-free enzyme assay. However, these compounds were ineffective in reducing HHQ production in a whole-cell *P. aeruginosa* assay. Ureidothiophene-2-carboxylic acid-based inhibitors were suggested to be expelled by efflux pumps in *P. aeruginosa*; if this were found to be the case, they would not be suitable for development as quorum-quenching strategies (Sahner et al., 2015). Based on the similarity between PqsD and the chalcone synthase (CHS2) expressed in alfalfa (*Medicago sativa*), Allegretta et al. (2015) developed new PqsD inhibitors from substrates of CHS2. These substrate analogs contained a catechol core that was important for inhibitory activity. Apparently, these compounds inhibited PqsD by blocking the enzyme substrate channel. Several of these inhibitors reduced the production of HHQ in bacterial cultures without affecting bacterial growth (Allegretta et al., 2015). Recently, Thomann et al. (2016) introduced an innovative and original strategy for quenching the PQS quorum-sensing system in *P. aeruginosa*. This strategy was based on the development of a dual-inhibitor compound that simultaneously inhibited both the PQS transcriptional regulator (PqsR) and PqsD. This compound acted as a dual inhibitor that affected the production of the virulence factors, pyocyanin and pyoverdine, but without affecting bacterial growth. Additionally, this compound reduced biofilm formation by *P. aeruginosa* and boosted the anti-bacterial activity of ciprofloxacin under biofilm conditions. Importantly, the dual inhibitor increased, in a dose-dependent manner and without cytotoxic effects, the survival rate of *G. mellonella* larvae challenged with lethal doses of *P. aeruginosa* (Thomann et al., 2016).

Inhibitors of the heterodimeric enzyme PqsBC have also been described. PqsBC participates in the PQS biosynthesis pathway by catalyzing the condensation of 2-aminobenzoyl acetate and octanoyl-CoA to form HHQ. Drees et al. (2016) demonstrated that 2-aminoacetophenone (secondary metabolite) acts as a competitive inhibitor of PqsBC and also inhibits HHQ production by *Pseudomonas putida* KT2440 (Drees et al., 2016). Moreover, PqsBC synthetic inhibitors more potent than 2-aminoacetophenone were recently described. This class of compounds were benzamide-benzimidazole derivatives and acted as dual inhibitors (acting simultaneously on PqsR and PqsBC). These PqsBC synthetic inhibitors attenuated *P. aeruginosa* PA14 virulence during infection of human lung epithelial cells and mouse macrophages. Some of the dual inhibitors reduced bacterial meropenem tolerance, specifically, the dual inhibitors with high anti-PqsR activity (Maura et al., 2017). Dual inhibitors with high anti-PqsR activity block 2-aminoacetophenone production more potently than dual inhibitors with low anti-PqsR activity, of particular interest because 2-aminoacetophenone has been associated with bacterial tolerance to antibiotics (Maura et al., 2017). Moreover, using two selective inhibitors to PqsBC, Allegretta et al. (2017) showed that in inhibitor-treated *P. aeruginosa* PA14 cells the 2-aminoacetophenone levels were higher than in non-treated bacteria. Consequently, the treatment with one of the PqsBC inhibitors favored *P. aeruginosa* PA14 tolerance to meropenem (Allegretta et al., 2017).

## Inhibition of Autoinducer Peptide Synthesis

In important Gram-positive pathogens including *S. aureus, Enteroccocus faecalis, Listeria monocytogenes, Clostridium difficile, Clostridium botulinum, Clostridium perfringens, Bacillus cereus, Streptococcus pyogenes* and others, the control of virulence factor expression is associated with peptide-based quorum sensing systems (Gray et al., 2013; Jimenez and Federle, 2014; Le and Otto, 2015; Singh et al., 2016; Ali et al., 2017). The *S. aureus* accessory gene regulator (*agr*) system and *E. faecalis fsr* quorum-sensing system are the most extensively characterized peptide-based quorum sensing systems (Gray et al., 2013; Ali et al., 2017; Tan et al., 2018).

The *S. aureus agr-*system controls the expression of several virulence factors, including RNAIII, δ-hemolysin, and phenol soluble modulins (PSMs). Transcription of the *agr* operon produces the RNA II and RNA III transcripts. Specifically, RNA II translation produces the proteins AgrA, AgrB, AgrC, and AgrD, which are the structural components of the *agr*-system, while RNAIII is involved in the post-transcriptional control of virulence factors expression and encodes δ-hemolysin (Tan et al., 2018). AgrD is the precursor of the autoinducer peptides (AIP) (AIP-I, AIP-II, AIP-III, and AIP-IV). AgrB is an endopeptidase involved in AIP maturation. The AgrB endopeptidase and the type I signal peptidase SpsB remove the C-terminal tail and N-terminal leader segment of AgrD, respectively, producing the thiolactone AIPs (LaSarre and Federle, 2013; Tan et al., 2018). The proteins AgrC/AgrA constitute a two-component system that is involved in AIP signaling. After AIPs are secreted, they bind to the histidine kinase AgrC receptor, which autophosphorylates with the subsequent transference of the phosphoryl group to the response regulator AgrA rendering phosphorylated AgrA. Phosphorylated AgrA forms a dimer, which at low concentration acts as a transcription factor that preferentially binds to the P2 promoter, triggering the production of RNAII transcripts. Consequently, the production of the *agr* components increases in an autocatalytic way (Wang and Muir, 2016; Tan et al., 2018). After phosphorylated AgrA accumulates to a threshold level, it binds to the P3 promoter stimulating the production of RNAIII transcripts. Additionally, phosphorylated AgrA binds to the *psm*α and *psm*β promoters, stimulating the production of PSMs (Gray et al., 2013; Tan et al., 2018). Because the *agr* system operates as a positively regulated auto-loop system, in principle, it is possible to disturb AIP production through interfering with any step of the circuit.

In a study performed by Kavanaugh et al. (2007), which focused on the identification of peptidases involved in AIP biosynthesis in *S. aureus*, the type I signal peptidase SpsB was identified as having a role in the *S. aureus* AIP biosynthesis pathway. Specifically, two SpsB inhibitors [(P+1) and NIF] were developed that consisted of peptides that mimic the N-terminal cleavage site of AgrD. The inhibitor NIF showed improved stability and stronger inhibition of quorum sensing in comparison to inhibitor P+1 (Kavanaugh et al., 2007). On the other hand, it has been reported that ambuic acid (a secondary fungal metabolite) inhibits the production of AIP in *S. aureus* as well as the biosynthesis of GBAP in *E. faecalis* and the putative cyclic peptide pheromones LsrD698 and LsrD826 in *Listeria innocua* (Nakayama et al., 2009). Later Todd et al. (2016), using an ultraperformance liquid chromatography coupled to mass spectrometry (UPLC-MS) platform, showed that ambuic acid suppresses AIP-I production by a clinical isolate of methicillin-resistant *S. aureus* (MRSA), in a dose-dependent manner (Todd et al., 2016). More recently, an MRSA strain was genetically manipulated to constitutively produce AIP-I without quorum-sensing control. Ambuic acid was found to effectively inhibit the biosynthesis of AIP-I by this strain. Interestingly, *in vivo* experiments in a murine model of intradermal MRSA challenge verified that ambuic acid attenuates MRSA pathogenesis and mediates quorum quenching *in vivo*. Moreover, ambuic acid proved effective in AIP biosynthesis inhibition of several pathogens besides *S. aureus*, i.e., *Staphylococcus saprophyticus, L. monocytogenes*, and *E. faecalis*, but did not affect commensal bacteria such as *Staphylococcus lugdunensis* and some *Staphylococcus epidermidis* strains. This selectivity is a desirable characteristic for therapeutic agents (Todd et al., 2017). In sum, all this evidence showed the potential of ambuic acid as an anti-virulence therapeutic agent.

Another target for the *agr*-system inhibition is the response regulator protein AgrA, which acts as *agr* operon transcriptional factor. The impairing of AgrA functionality might perturb the *agr* operon transcription and consequently AIP production as well as *agr*-controlled virulence factors production. Several chemical compounds including 2-(4-methylphenyl)-1,3-thiazole-4-carboxylic acid, 9H-xanthene-9-carboxylic acid, 4-phenoxyphenol, savirin, ω-hydroxyemodin, biaryl hydroxyketones, and norlichexanthone appear to act by blocking the binding of AgrA to the *agr* operon promoters via direct interaction with the AgrA C-terminal DNA binding domain (Leonard et al., 2012; Sully et al., 2014; Daly et al., 2015; Baldry et al., 2016; Greenberg et al., 2018). Moreover, other compounds like naphthalene derivatives and biaryl compounds potentially could bind to the AgrA N-terminal phosphoryl-binding pocket, interfering with ArgA phosphorylation and binding to DNA (Khodaverdian et al., 2013).

Recently, some of these AgrA inhibitors have shown be promising in attenuating bacterial virulence *in vivo*. In this regard, ω-hydroxyemodin (a polyhidroxyanthraquinone) inhibited *in vitro* all the *S. aureus* agr-system types (I–IV). Consequently, ω-hydroxyemodin treatment reduced the RNAIII*, psm*α and *hla* transcription without bactericidal and cytotoxic-associated effects. In addition, ω-hydroxyemodin inhibited the *S. epidermidis agr*-system and attenuated the virulence of *S. aureus* in a mouse skin and soft tissue infection model, apparently via disruption of the *arg*-system, facilitating the bacterial clearance by the host immune system (Daly et al., 2015). Previously, Sully et al. (2014), using an airpouch skin infection model and a dermonecrosis model, described *S. aureus*-attenuated virulence by savirin via *agr*-system disruption and improved host immune response. The treatment of *S. aureus* USA300 strain LAC with savirin down-regulated the expression of several *agr*-regulated virulence factors, including RNAIII, V8 protease, serine proteases, lipase, staphopain, PMSβ1, PMSα, PVL, and others, whereas it up-regulated the expression of Spa, SdrD and fibrinogen-binding protein. In addition, savirin treatment reduced α-hemolysin, protease and lipase activity. Clinical isolates (comprising the *agr*-systems I, II, III, and IV) treated with savirin down-regulated *psm*α transcript levels, and α-hemolysin activity was reduced in several MRSA and MSSA clinical isolates. Interestingly, resistance or tolerance to savirin were not observed, and the *S. epidermidis agr* system was not significantly disturbed (Sully et al., 2014). Moreover, two biaryl hydroxyketones (F12 and F19) have been reported that downregulated *hla, psm*α and RNAIII expression in the MRSA USA300 strain. The methicillin-resistant *Staphylococcus epidermidis* (MRSE) strain treated with F19 down-regulated AtlE, psmα and RNAIII transcript levels. F-19 protected monocyte and macrophage cells from the lysis caused by several Gram-positive pathogens. Importantly, in an MRSA wound infection model, compound F-19 potentiated β-lactam and fluoroquinolone antibiotic activity, whereas in an MRSA bacteremia/sepsis model, F-19 alone and in combination with cephalothin protected the animals from a lethal infection with MRSA (Greenberg et al., 2018). In a previous study, F-12 and F-19 treatment increased the survival time of MRSA-infected larvae, as well as when they were used in combination with β-lactam antibiotics. In addition, in mice, F-12 stimulated the healing of MRSA-infected wounds (Kuo et al., 2015). Antisense oligonucleotides that target the *agrA* mRNA have also been used to inhibit AgrA activity. Recently, antisense oligonucleotides against *agrA* mRNA were used as a strategy to quench the *agr*-system in a community-associated MRSA strain (CA-MRSA USA300 LAC). The antisense oligonucleotide treatment affected the *agrA* expression as well as the expression of several virulence factors, including *psm*α, *psm*β, *hla*, and *pvl*. The CA-MRSA USA300 LAC strain virulence in a mouse subcutaneous infection model was attenuated by antisense oligonucleotide treatment (Da et al., 2017).

Disruption of the *agr*-system via interference with AgrC activity, in principle, could also influence AIP biosynthesis. The most exploited strategy to develop AgrC inhibitors is based on producing structural modifications in native AIP scaffold to yield AIP structural analogs (Singh et al., 2016; Wang and Muir, 2016). Some of the recently developed AgrC inhibitors are amide-bridged AIP-III analogs, in which the thioester bond was replaced by an amide bond conferring higher hydrolytic stability and solubility in aqueous media on them than their precursors. The introduction of the amide bridge did not severely affect the inhibitory potency of the lactam analogs toward *S. aureus* AgrC (type I-IV) (Tal-Gan et al., 2016). Simplified AIP-II peptidomimetics were developed from a truncated AIP-II by Vasquez et al. (2017). Some of these peptidomimetics were pan-group *S. aureus* AgrC inhibitors; however, the most soluble mimetic in aqueous media (a desirable characteristic for the inhibitors) did not show a potent inhibitory activity toward *S. aureus* AgrC (group III-IV) in comparison with the parental peptide (truncated AIP-II), but displayed an inhibitory activity similar to the parental peptide toward *S. aureus* group I, which is one of the main etiologic agents in human infections (Vasquez et al., 2017). Moreover, Karathanasi et al. (2018) described linear synthetic peptidomimetics that interfered with the *S. aureus agr*-system through competitive binding to the AgrC receptor (Karathanasi et al., 2018). Recently, other AgrC inhibitors described were *S. epidermidis* AIP and *S. lugdunensis* AIP analogs (Gordon et al., 2016; Yang et al., 2016). Furthermore, secondary metabolites such as WS9326A, WS9326B, and cochimicin II/III from actinomycetes, avellanin from *Hamigera ingelheimensis*, ngercheumicins and solonamides from *Photobacterium* sp. strain S2753 probably influence the AgrC activity via competitive inhibition (Mansson et al., 2011; Kjaerulff et al., 2013; Desouky et al., 2015; Igarashi et al., 2015; Wang and Muir, 2016). Solonamide B showed to be effective in a mouse model for atopic dermatitis to attenuate *S. aureus* virulence via δ-toxin-induced immunopathologic response inhibition (Baldry et al., 2018).

## Inhibition of N-Acyl-Homoserine Lactone Synthesis

In Gram-negative bacteria, quorum-sensing systems based on acyl-HSLs as signal molecules are the most common. The category of acyl-HSLs (also known as autoinducer-1) comprises more than 30 different molecules that share a common structural scaffold, consisting of an acyl chain linked to a homoserine lactone ring. The acyl chains vary in length (4-18 carbons), oxidation state, and degree of saturation (LaSarre and Federle, 2013; Chan et al., 2015). The acyl-HSLs are biosynthesized mainly by the acyl-HSL synthases belonging to the Lux I family (Lux I-type acyl-HSL synthases). These synthases use as substrates S-adenosyl-L-methionine (SAM) and acylated acyl-carrier protein (acyl-ACP) and yield the respective acyl-HSL, the holo-ACP, and MTA, as products (Chung et al., 2011). The Lux I-type acyl-HSLs synthases are present in hundreds of bacterial species, and enzymes from different bacterial species may share conserved regions. Lux I-type acyl-HSLs synthases are not present in Eukarya, making them a potential target for the development of quorum-sensing inhibitors (Chan et al., 2015; Papenfort and Bassler, 2016).

The synthesis of butyryl-HSL is mediated by *P. aeruginosa* RhlI synthase using as substrates butyryl-ACP and SAM. An early study by Parsek et al. (1999) showed that the end products, MTA, and holo-ACP, and the SAM substrate analogs SAH, S-adenosylcysteine, and sinefungin, act as RhlI synthase inhibitors (Parsek et al., 1999). Another acyl-HSL synthase present in *P. aeruginosa* for which inhibitors have been reported is LasI. In a study by Lidor et al. (2015), the compound (z)-5-octylidenethiazolidine-2, 4-dione (TZD-C8) was found to inhibit biofilm formation by *P. aeruginosa* PAO1 in a dose-dependent manner, as well as to induce the downregulation of the expression of the *pqsABCDE* operon and the *lasI* gene. Therefore, potentially TZD-C8 could perturb both the quorum-sensing system based on PQS and that based on 3-oxo-C12-HSL. The *in vitro* swarming motility and PQS production of the bacteria were also affected. *In silico* evaluation of the interaction between TZD-C8 and LasI suggested that the inhibitory activity of TZD-C8 could result from its binding to the LasI activity pocket (Lidor et al., 2015). In the Gram-negative bacterium *Burkholderia glumae* the quorum-sensing signal octanoyl-L-HSL (C8-HSL) is synthesized by the acyl-HSL synthase TofI. Chung et al. (2011) found the TofI inhibitor J8-C8 from a library of acyl-HSL analogs. This compound reduced the production of C8-HSL by *B. glumae* BGR1 cells. In addition, J8-C8 inhibited C8-HSL synthesis in a dose-dependent manner and, together with MTA, had a synergistic inhibitory effect on TofI. X-ray crystal structure analyses showed that J8-C8 binds to the acyl-ACP binding site on TofI, specifically the binding site for the acyl chain, while MTA independently binds to the binding site for SAM (Chung et al., 2011). Moreover, Christensen et al. (2013) reported five compounds that inhibited *Burkholderia mallei* BmaI1 synthase and YspI synthase from *Y. pestis*, which is phylogenetically distant from *B. mallei* BmaI1 synthase. Additionally, two of the five compounds were found to reduce the production of octanoyl-HSL without affecting bacterial growth. The most potent compound [3-(4-methylpiperazin-1-yl)(pyridin-2-yl)methyl-2-phenyl-1H-indol-1-ol] acted as a noncompetitive inhibitor of BmaI1 synthase, and some analogs of this compound showed inhibitory activity. One interesting finding in this study was that one of the five inhibitory compounds selected was the cephalosporin antibiotic cefatrizine, suggesting that cephalosporin antibiotics may inhibit acyl-HSL synthases (Christensen et al., 2013).

It has been reported that thioether analogs of the thioester acyl-substrates of acyl-HSL synthase inhibit the enzyme. Specifically, octyl-ACP noncompetitively inhibited *B. mallei* BmaI1 synthase while the isopentyl-CoA competitively inhibited the *Bradyrhizobium japonicum* BjaI-synthase (Christensen et al., 2014). Recently, new diketopiperazine derivatives have been described that inhibit *Burkholderia cenocepacia* CepI acyl-HSL synthase. The most potent of these derivatives [(3S)-3-Benzyl-6-(3,6-dioxocyclohexa-1,4-dien-1-yl)piperazine-2,5-dione] acted as a non-competitive inhibitor toward both C8-ACP and SAM substrates. This finding was also supported by molecular docking analysis, which showed several high-affinity contact sites for the inhibitor on the CepI structure, but none of these sites was the SAM- and acyl substrate-binding site. Besides, some of these diketopiperazine derivative compounds did not exert an antimicrobial effect on *B. cenocepacia* J2315 but did interfere with the production of virulence factors such as proteases and siderophores. Furthermore, they perturbed biofilm formation, protected *Caenorhabditis elegans* nematodes against infection with *B. cenocepacia* J2315, and had low toxicity for HeLa cells (Scoffone et al., 2016). Recently, based on comparative proteomics approach, Buroni et al. (2018) demonstrated that *B. cenocepacia* J2315 treated with (3S)-3-benzyl-6-(3,6-dioxocyclohexa-1,4-dien-1-yl)piperazine-2,5-dione displayed a protein expression pattern quite similar to *B. cenocepacia* Δ*cepI* mutant (*B. cenocepacia* J2315 with deleted *cepI* gene). Interestingly, both the inhibitor-treated strain and the Δ*cepI* mutant overexpressed the giant cable pilus protein CblA, which has been associated with *B. cenocepacia* virulence *in vivo*. In addition, using site-directed mutagenesis and enzymatic activity inhibition approaches it was observed that the pocket around the Ser 41 residue on the CepI structure appears to be the inhibitor binding site (Buroni et al., 2018).

Quorum-quenching agents of plant origin have been identified as inhibitors of acyl-HSL synthases. Chang et al. (2014) identified salicylic acid, tannic acid, and trans-cinnamaldehyde as potential acyl-HSL synthase inhibitors. Subsequently, it was demonstrated that trans-cinnamaldehyde was an RhlI-specific inhibitor and did not affect the growth of *P. aeruginosa*. Molecular docking analysis of trans-cinnamaldehyde suggested that this inhibition might be mediated by the occupation of the substrate-binding pocket on the synthase (Chang et al., 2014). Recently, other plant-derived compounds have been identified as acyl-HSL synthase inhibitors. It was observed that carvacrol and eugenol reduce biofilm formation, the activity of plant cell wall degrading enzymes, the expression of quorum-sensing-related genes, and virulence in the phytopathogens *Pectobacterium carotovorum* subsp*. brasiliense* Pcb1692 and *Pectobacterium aroidearum* PC1. Based on docking of these compounds to the computational models of ExpR (regulatory protein) and ExpI (acyl-HSL synthase), the mechanism of action of these quorum-quenching agents was suggested to involve direct interaction with ExpI/ExpR proteins and the consequent inhibition of acyl-HSL production (Joshi et al., 2016).

Even though most of the strategies for inhibiting signal production have targeted the acyl-HSL synthases, it is possible that other enzymes linked to the signal biosynthesis pathway could also be targeted. In this respect, it has been reported that triclosan inhibited the *P. aeruginosa* enoyl-acyl carrier protein reductase (FabI) *in vitro*. This inhibition reduced the production of butyryl-HSL because FabI supplies the butyryl-ACP necessary for RhlI synthase-mediated butyryl-HSL synthesis (Hoang and Schweizer, 1999). Since FabI is involved in the metabolism of the fatty acids (an essential process for the bacteria), inhibitory agents directed toward it could potentially exert selective pressure on the bacteria with the subsequent emergence of resistant mutants. In fact, resistance to triclosan by *P. aeruginosa* PAO1 has been reported; this resistance results from active efflux pumps and a triclosan-resistant enoyl-acyl carrier protein reductase (FabV) (Chuanchuen et al., 2003; Zhu et al., 2010; Huang et al., 2016).

## Future Directions

The book “*Sun Tzu on The Art of War”* postulated that “*In the practical art of war, the best thing of all is to take the enemy's country whole and intact; to shatter and destroy it is not so good. So, too, it is better to recapture an army entire than to destroy it, to capture a regiment, a detachment or a company entire than to destroy them*” (Giles, 2000). In this light, the war of science against bacterial pathogens should not exclusively focus on novel bactericidal agents (which would destroy the enemy), but should also consider antivirulence factors (which would trap the enemy, leaving it without weapons and/or communication systems), allowing the human or animal host to subsequently eliminate the pathogens. Because quorum sensing is a critical process for controlling collective traits including lifestyle and biofilm formation, the synthetic modulators of quorum sensing seem to be the key to manipulating bacterial behavior on demand. This is particularly so in the case of pathogenic bacteria, whose virulence factors include quorum sensing mechanisms (Papenfort and Bassler, 2016).

In this Review, we have summarized the main targets for quorum sensing signal biosynthesis inhibition. The control of bacterial behavior by small molecules has been viewed as a promising strategy for the control of biofilms; and despite the differences among species, quorum sensing plays a crucial role in the infectious process. Although therapies that affect quorum sensing are less likely to select for resistance in comparison with traditional antibiotics, some cases reported in the literature show that bacteria can become resistant to quorum-sensing inhibitors (Defoirdt et al., 2010; Kalia et al., 2014; García-Contreras et al., 2016). Nevertheless, the selective pressure exerted by traditional antibiotics is higher than that of the quorum-sensing inhibitors; therefore, the latter may have longer functional lives and greater utility in treating bacterial infections than the former, which have been, in many cases, rendered ineffective by resistance. To date, few clinical trials of molecules that inhibit quorum sensing have been conducted (Papenfort and Bassler, 2016); therefore, it is still too early to assess the therapeutic potential of these molecules. Efforts to determine mechanisms of resistance and to screen for more effective inhibitors, as well as studies focusing on the *in vivo* application of such molecules, could lead to the next generation of antimicrobial agents.

## Author Contributions

OF, OS, CFN, and OLF contributed conception and design of the review. OF, PR, ÁP, WP, and OS wrote the manuscript. PR and ÁP made the figures. OLF and CFN revised the manuscript. All authors read and approved the submitted version.

### Conflict of Interest Statement

The authors declare that the research was conducted in the absence of any commercial or financial relationships that could be construed as a potential conflict of interest.
